# Machine learning screening of bile acid-binding peptides in a peptide database derived from food proteins

**DOI:** 10.1038/s41598-021-95461-1

**Published:** 2021-08-09

**Authors:** Kento Imai, Kazunori Shimizu, Hiroyuki Honda

**Affiliations:** 1 Department of Biomolecular Engineering, Graduate School of Engineering, Nagoya University, Nagoya, 464-8603 Japan; 2 Japan Society for the Promotion of Science, Research Fellowship for Young Scientists, Chiyoda-ku, Tokyo, Japan

**Keywords:** Biochemistry, Health care

## Abstract

Bioactive peptides (BPs) are protein fragments that exhibit a wide variety of physicochemical properties, such as basic, acidic, hydrophobic, and hydrophilic properties; thus, they have the potential to interact with a variety of biomolecules, whereas neither carbohydrates nor fatty acids have such diverse properties. Therefore, BP is considered to be a new generation of biologically active regulators. Recently, some BPs that have shown positive benefits in humans have been screened from edible proteins. In the present study, a new BP screening method was developed using BIOPEP-UWM and machine learning. Training data were initially obtained using high-throughput techniques, and positive and negative datasets were generated. The predictive model was generated by calculating the explanatory variables of the peptides. To understand both site-specific and global characteristics, amino acid features (for site-specific characteristics) and peptide global features (for global characteristics) were generated. The constructed models were applied to the peptide database generated using BIOPEP-UWM, and bioactivity was predicted to explore candidate bile acid-binding peptides. Using this strategy, seven novel bile acid-binding peptides (VFWM, QRIFW, RVWVQ, LIRYTK, NGDEPL, PTFTRKL, and KISQRYQ) were identified. Our novel screening method can be easily applied to industrial applications using whole edible proteins. The proposed approach would be useful for identifying bile acid-binding peptides, as well as other BPs, as long as a large amount of training data can be obtained.

## Introduction

Bioactive peptides (BPs) are protein fragments that have positive benefits in humans^1^. BPs exhibit a wide variety of physicochemical properties, such as basic, acidic, hydrophobic, and hydrophilic properties. Therefore, they have the potential to interact with a variety of biomolecules, whereas neither carbohydrates nor fatty acids have such diverse properties. Therefore, BPs are considered a new generation of biologically active regulators^2^ and are promising candidates for the cosmetic and health food industry. Recently, some BPs have been screened from edible proteins^3,4^. For example, the alpha-casein-derived peptides RYLGY, AYFYPEL, and YQKFPQY have angiotensin-converting enzyme (ACE)-inhibitory activity^5^, and the beta-lactoglobulin-derived peptides VAGTWY, AASDISLLDAQSAPLR, IPAVFK, and VLVLDTDYK have bactericidal activity^6^.

Current advanced approaches to peptide screening have been reported by some research groups. In particular, directed evolution is a promising methodology. Gray et al*.* reported the evolution of macrocyclic peptides by scanning unusual protease resistant mRNA displays and discovered MX8K cyclic peptides targeting the autophagy protein LC3^7^. Navaratna et al*.* reported the stabilization of peptide evolution by *E. coli* displays^8^. Peptide stabilization was performed by click chemistry using bis-alkyne molecules, and stabilized peptides showed 4–9 times higher affinity and high protease stability. However, novel BP fragments from edible proteins have not yet been discovered.

Peptide screening from edible proteins remains a difficult task. The vast majority of BPs are encrypted in the structure of the parent proteins and are released mainly by enzymatic processes. BPs are present in complex matrices containing a large number of hydrolyzed protein fractions; therefore, it is necessary to separate and purify them^4^. Until now, BP identification has been conducted with a trial-and-error approach, including selection of food materials and enzymes, separation of the BP fraction by liquid chromatography (LC), extraction from other materials, and concentration of BP. In addition, in many cases, the initial proteolytic mixture is prepared based on the specific interests of researchers and industries, with no a priori knowledge, and no guarantee that the desired BPs are present. The common approach therefore has a significant ‘trial and error’ element, potentially leading to wasted time and money.

In silico approaches for identifying novel BPs have been proposed^9,10^. In silico approaches make use of peptide databases containing sequences derived from proteins of interest and implement bioinformatic tools to predict bioactivity. Many peptide databases have been developed, including the database BIOPEP-UWM^11^, which stores BPs along with edible proteins, allergenic proteins with their epitopes and sensory peptides, and amino acids. In addition, it implements predictive tools, including the theoretical degree of hydrolysis and bioactivity prediction. Using the BIOPEP-UWM database, the appropriate fraction of DPP-4 (dipeptidyl peptidase-4) inhibiting peptides derived from mealworms (*Tenebrio molitor*) was selected^12^. This approach has also been adopted in pigeon pea (*Cajanus cajan*)^13^. Recent studies have shown that the combination of databases with advanced machine-learning-based bioinformatics tools is a promising approach for screening and developing novel BPs. For example, Meher et al*.*^14^ created an antimicrobial peptide by using predictive models with support vector machine (SVM) algorithms and antimicrobial databases CAMP^15^, APD3^16^ and AntiBP2^17^. Gautam et al*.* predicted cell-penetrating activity by using SVM and novel databases^18^ and achieved a maximum accuracy of 97.4%.

**Figure 1:**
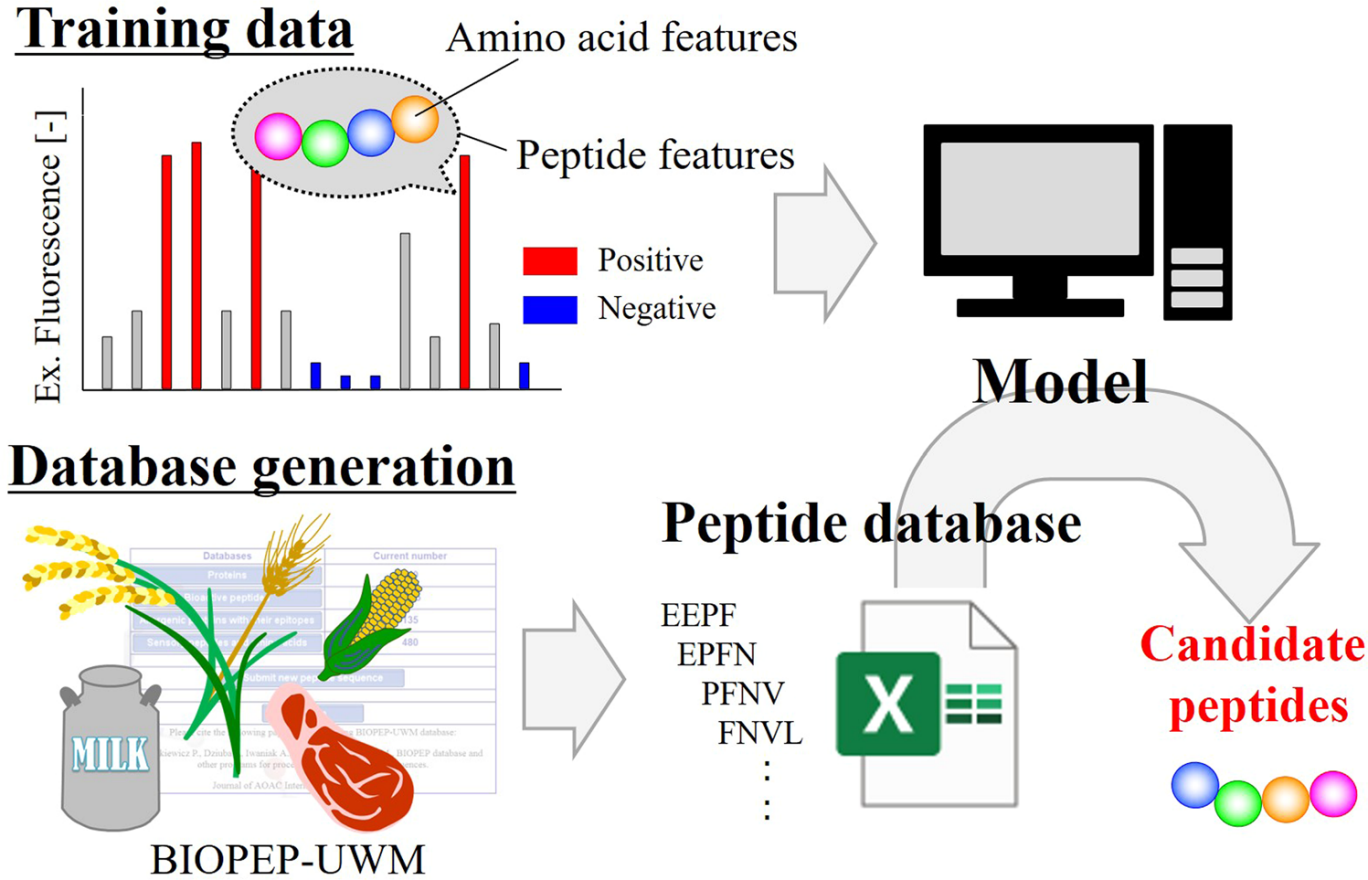
Schematic showing experimental workflow. Positive and negative datasets were generated from the training data. Subsequently, explanatory variables were generated with amino acid features (site-specific features) and peptide features (global features). Predictive models were constructed using a combination of the training data and explanatory variables. A new database containing peptides found in edible proteins was created using the edible protein database BIOPEP-UWM. Lastly, constructed models were applied to the edible protein database and the bioactivity of each peptide was predicted.

In the present study, a novel strategy to screen BPs derived from edible proteins was developed using BIOPEP-UWM and machine learning. Machine learning using training data is convenient for acquiring the sequence characteristics of BPs. If the acquired model has high prediction accuracy, the derived BP fragments can be predicted without any wet experiment. This strategy allows for the exploration of all BPs from edible proteins by in silico screening using databases such as BIOPEP-UWM. The experimental workflow is shown in Fig. 1. We used the training data obtained with a high-throughput peptide array to generate positive and negative datasets. The predictive model was generated using the explanatory variables of the peptides in these datasets. Finally, this model was applied to a peptide database derived from edible proteins using BIOPEP-UWM. In the method proposed here, the desired BP was identified first by the machine-learning method, and then the food materials were selected. It should be noted that optimization of the separation process was established without a trial-and-error approach after BP has been chemically synthesized. This is the opposite or reverse approach for BP identification. The aim of this study was to demonstrate the proof-of-concept that BP can be identified from a large number of candidate proteins, by using the opposite approach.

We tested our peptide screening tool by searching for bile acid-binding peptides. In humans, cholesterol absorption occurs in the proximal jejunum of the small intestine, where both dietary cholesterol and biliary cholesterol are available for uptake from the intestinal lumen via bile acid micelles^19,20^. Bile acid-binding peptides interact with bile acids that form micelles and subsequently disrupt the micelles, contributing to the suppression of intestinal cholesterol absorption. We previously designed bile acid-binding peptides using an informatics approach^21–24^. However, the designed peptides were not found in storage proteins or protein sources, and proteases were selected based on our interests. Bile acid-binding peptides work on the intestinal tract, and we therefore do not need to consider their absorption from the small intestine when developing novel health foods. Using our novel approach, we have established a framework for rapid and cost-effective screening of BPs, which may be applied to the development of new health-promoting products.

## Results and discussion

### Measurement of bile acid binding in a synthetic peptide array

**Figure 2:**
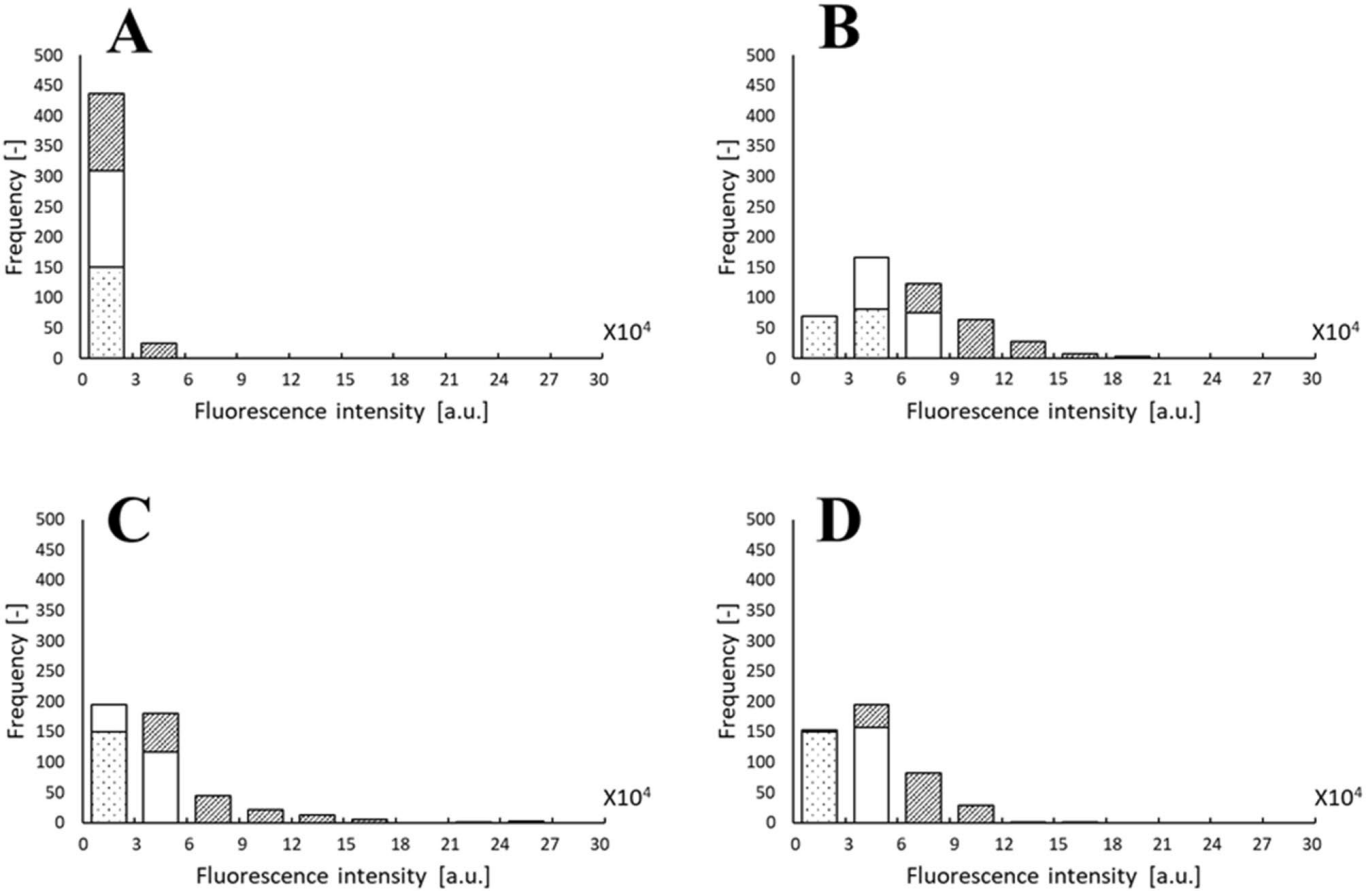
Frequency distributions of fluorescent intensities of all peptides such as 150 positive (slashed bar), 150 negative (dotted bar) and others (blank bar). (**A**) 4-mer, (**B**) 5-mer, (**C**) 6-mer, (**D**) 7-mer.

Training data are essential for the construction of classification models. To generate training data, 460 4-, 5-, 6-, and 7-mer peptides were chemically synthesized in the peptide array. A part of the synthesized peptide was identified by MS analysis to verify that the synthesized peptides coincided with the designed amino acid sequences. Assessment of binding ability between bile acid and peptides was performed using two kinds of antibodies: a first antibody against bile acid and a fluorescent-labeled secondary antibody. As a binding activity of the peptide, the average fluorescence intensities were determined based on the triplicate fluorescence intensities of the same peptide sequence. The sequences and fluorescent intensities are shown in Supplementary Table S2 and Supplementary Figs. S1–S6. The fluorescence intensity of 4-mers was lower than that of longer peptides (Supplementary Fig. S2A). The observed low intensity of 4-mers in the training data may be due to the relatively low hydrophobicity of the 4-mer peptides. Using the peptide array data, 150 peptides with the highest fluorescent intensities were defined as the ‘positive’ dataset, and 150 peptides with the lowest fluorescent intensities were defined as a ‘negative’ dataset for bile acid binding activity. The average fluorescence intensities of the positive and negative datasets are presented in Table 1. Here, 150 positive dataset numbers were selected because the average fluorescence intensities of positive datasets were more than the average plus SD of 460 peptides. The same number of negative datasets were selected. The frequency distributions of the fluorescent intensities of all peptides are shown in Fig. 2 for clarity. The distribution of the 5-mer was slightly broader than that of the others. When the peptides became longer, the hydrophobicity of the peptide gradually increased. This may be the reason the high performance of 5-mer was obtained, as shown in Table 2. Since there was a significant difference between the two datasets (P < 0.001), the randomly designed peptide library contained peptides with different bile acid-binding bioactivities.

**Table 1:** Average of the fluorescence intensities of top 150 positive and bottom 150 negative training datasets, based on the rank of fluorescent intensities.

Residue	4-mer	5-mer	6-mer	7-mer
Average	17,823	64,761	43,677	44,235
SD	7824	35,329	34,027	25,330
Positive	26525 ± 5820	106731 ± 24476	79019 ± 39473	73980 ± 18873
Negative	9959 ± 3380	29325 ± 7769	19202 ± 4362	19108 ± 6793

**Table 2:** The predictive scores of each prediction algorithm for identifying peptides with acid bile binding activity. SVF: support vector machine, RF: random forest, LR: logistic regression.

	Accuracy	Precision	Recall
**4-mer**			
SVM	0.667	0.664	0.673
RF	0.757	0.720	0.840
LR	0.693	0.691	0.700
**5-mer**			
SVM	0.943	0.940	0.947
RF	0.953	0.936	0.973
LR	0.887	0.877	0.900
**6-mer**			
SVM	0.880	0.896	0.860
RF	0.900	0.905	0.893
LR	0.863	0.866	0.860
**7-mer**			
SVM	0.883	0.876	0.893
RF	0.897	0.870	0.933
LR	0.897	0.884	0.913

### Construction of predictive model and evaluation of model performance

To construct the predictive model, 7 amino acid features (AAF), including isoelectric point (IP), polarity (PL), hydropathy index (HI), molecular weight (MW), index of helix (Ph), and index of turn (Pt) listed in Supplementary Table S1 were selected. The collinearity of these features is contradictory. Since these were used as site-specific characteristics, the number of these AAFs was 28 for 4-mer, 35 for 5-mer, 42 for 6-mer, and 49 for 7-mer peptides. Next, the characteristics of peptides, not amino acids, were generated using these seven AAFs. The average of each AAF was generated as important global feature (GF). However, even though similar averages of an AAF such as IP are nominated in two arbitrary peptides, the peptide features are quite different if the maximum or minimum of the AAF of peptides differ. For instance, even though the average of IP is neutral, it is considered that the features of peptides consisting of non-charged amino acids are quite different from those of peptides consisting of positively and negatively charged amino acids. To explain the peptide feature, therefore, the deviation of AAF, the maximum, and the minimum were generated as GFs. Since there are seven AAFs, 28 GFs were generated independent of peptide length.

To perform machine learning, peptide features such as AAFs and GFs of 300 peptides (positive = 150, negative = 150) were calculated. For each 4-mer, 56 features were generated (28 AAFs and 28 GFs), for each 5-mer 63 (35 AAFs and 28 GFs), 6-mer 70 (42 AAFs and 28 GFs), and 7-mer 77 (49 AAFs and 28 GFs), and used as explanatory variables. Three algorithms were used to construct the predictive model (SVM, RF, and LR), and the model performance was evaluated by comparing the accuracy, precision, and recall. Peptides with a probability of > 0.5, designated as positive, and those with a probability of < 0.5, were designated as negative for bile acid binding ability. Except for the precision scores of 5- and 7-mers, all RF scores were the highest among the three tested algorithms (Table 2). Therefore, RF was selected as the predictive algorithm.

The scores 4-mer peptides were lower than the scores of longer peptides (Table 2). The ratio of the average fluorescence intensity of positive the dataset and that of the negative dataset was defined as the P/N intensity ratio. In Table 1, the P/N intensity ratio of 4-mers (2.67) was lower than that of longer peptides (3.63 for 5-mers, 4.11 for 6-mers, 3.87 for 7-mers). This is caused by the relatively lower overall fluorescence intensity of the 4-mer training data. The model performance was roughly corelated with the P/N intensity ratio. The reason for the poor performance is the relatively large number of FPs and FNs predicted by the acquired model when the P/N intensity ratio is low.

In order to predict the bioactivity of peptides, quantitative analysis of the relationship between the structure and bioactivity of peptides has received much interest from many physical biochemists. In a recent study^25^, the hydrophobicity of the amino acid located at the N-terminal end was reported to be more hydrophilic than that of the same amino acid located at both the middle and C-terminal ends. Therefore, it is likely that 4-mer peptides are more hydrophilic than longer peptides, such as 5-, 6-, and 7-mer peptides. The reason why 4-mer peptides show lower binding to bile acid is also considerable because of the lower hydrophobic interaction between the 4-mer peptide and bile acid. However, hydrophobicity is necessary for the strong binding of peptides to bile acid. In our previous paper, we identified bile acid-binding 4-mer peptides such as NGLK, YEAR, etc.^21^. These peptides showed similar or higher binding activity compared to the 6-mer binding peptides. It is likely that the 4-mer binding peptides show different physiochemical features compared with those of longer peptides.

To investigate the importance of the input features, the variable importance was estimated according to the increase in the predictive error due to the permutation of out-of-bag data for the given variable. The importance of each input variable is listed in Supplementary Table S3. Most of the top 10 selected features referred to the GFs of peptides, namely av, sd, min, max, with the exception of two specific features: residue2_Molecular_weight for 4-mers and residue1_Isoelectric_point for 7-mers. In addition, two features for 4-mers, four features for 5-mers, four features for 6-mers, and five features for 7-mers were related to the peptide isoelectric point. Similarly, five features for 4-mers, three features for 5-mers, two features for 6-mers, and two features for 7-mers were related to molecular weight. This suggests that the GFs are more important than the site-specific features for bile acid-binding activity in peptides of 4–7 amino acids. Bile acid molecules are amphiphilic, with a hydrophobic steroid core and hydrophilic hydroxyl groups, and therefore, have strong surfactant action. Since peptide binding can occur in either direction with bile acids, site-specific peptide features may be less important.

Features referring to the isoelectric point and molecular weight are among the most important in Supplementary Table S3. This suggests that peptides with high isoelectric points or high molecular weights bind strongly to bile acids. The five amino acids with the highest isoelectric points were R, K, H, P, and I^26^, and the top five for molecular weight were W, Y, R, F, and H^27^. Therefore, basic or aromatic peptides have higher binding activity against bile acids. Some studies have investigated the binding mechanisms between bile acids and other compounds, such as sterols and nisin^28–31^, and revealed that hydrophobic amino acids, especially aromatic amino acids, interact with bile acid micelles. These findings are in agreement with the top 10 features listed in Supplementary Table S3.

**Figure 3:**
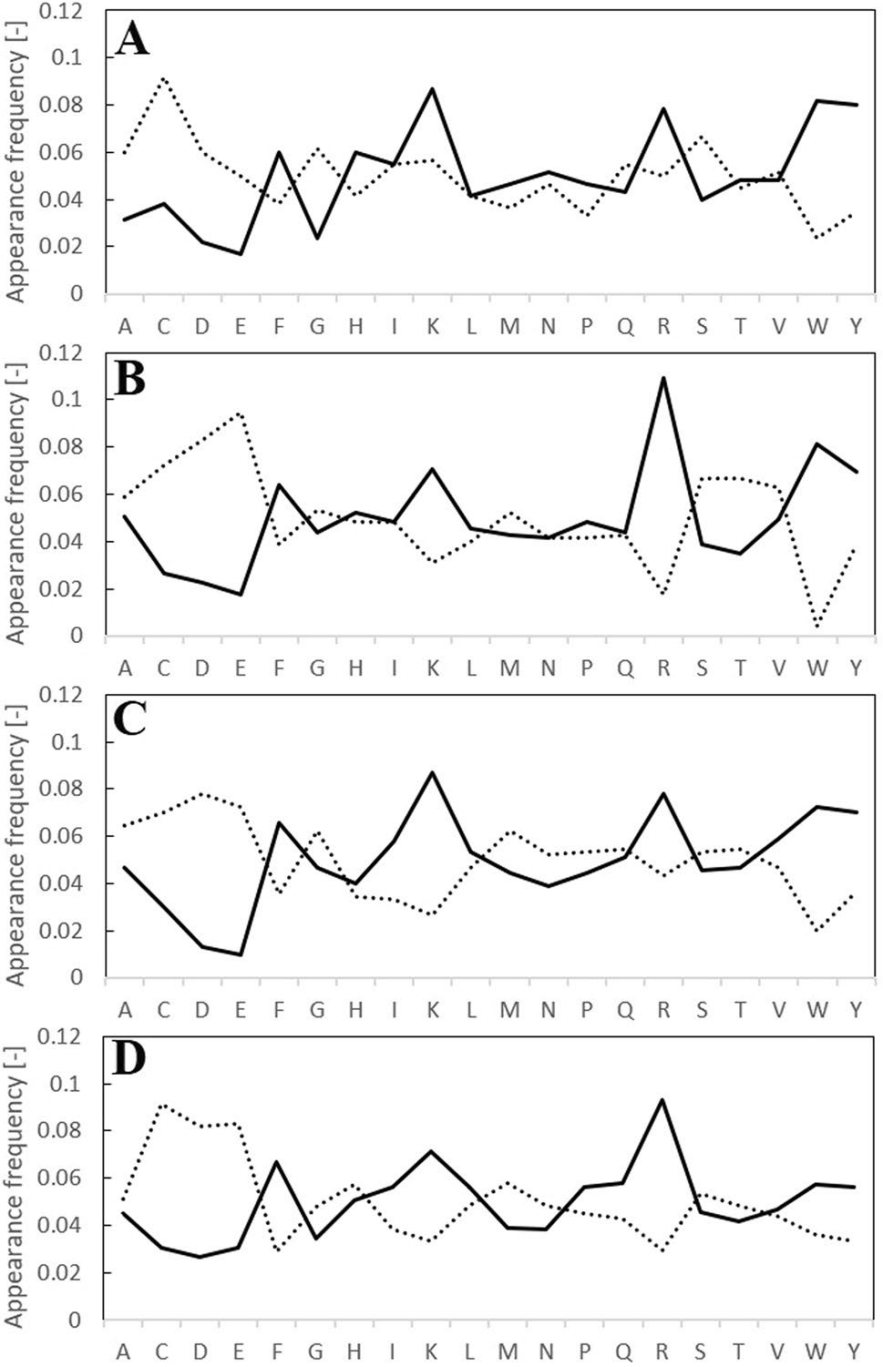
Appearance frequency of amino acid residues for 150 positive (solid lines) and 150 negative (dotted lines) peptides [(**A**) 4-mer, (**B**) 5-mer, (**C**) 6-mer, (**D**) 7-mer].

We analyzed the appearance frequency of amino acid residues for peptides listed in Supplementary Table S2 and obtained Fig. 3 to verify the reproducibility of the learning data. In the appearance frequency of amino acids for positive peptides, 5 amino acids, F, K, R, W, and Y, showed high frequency. Among the negative peptides, 3 amino acids, C, D, and E, were relatively high. These results coincided with the results of the feature analysis from Supplementary Table S3. However, in the case of 4-mer peptides, a slightly different frequency was obtained; A and G were relatively low in positive peptides while D and E were relatively low in negative peptides.

### Construction of edible peptide database and prediction of bile acid binding activities

A set of 710 edible proteins were obtained from BIOPEP-UWM and digested using all available predicted protease binding sites (Table 3), resulting in 199568 4-mers, 198808 5-mers, 198055 6-mers, and 197310 7-mers. After removing duplicate sequences, the dataset contained 56171 4-mers, 89663 5-mers, 98387 6-mers, and 102805 7-mers. Thus, a total dataset of approximately 350000 peptide sequences was generated.

**Table 3:** The numbers of peptides derived from edible proteins by performing in silico protease digestion using all available proteases in the database.

Residue	Number of fragments	Number of unique fragments
4-mer	199,568	56,171
5-mer	198,808	89,663
6-mer	198,055	98,387
7-mer	197,310	102,805

After removing duplicate sequences, the final number of peptides is shown in the right column.

**Figure 4:**
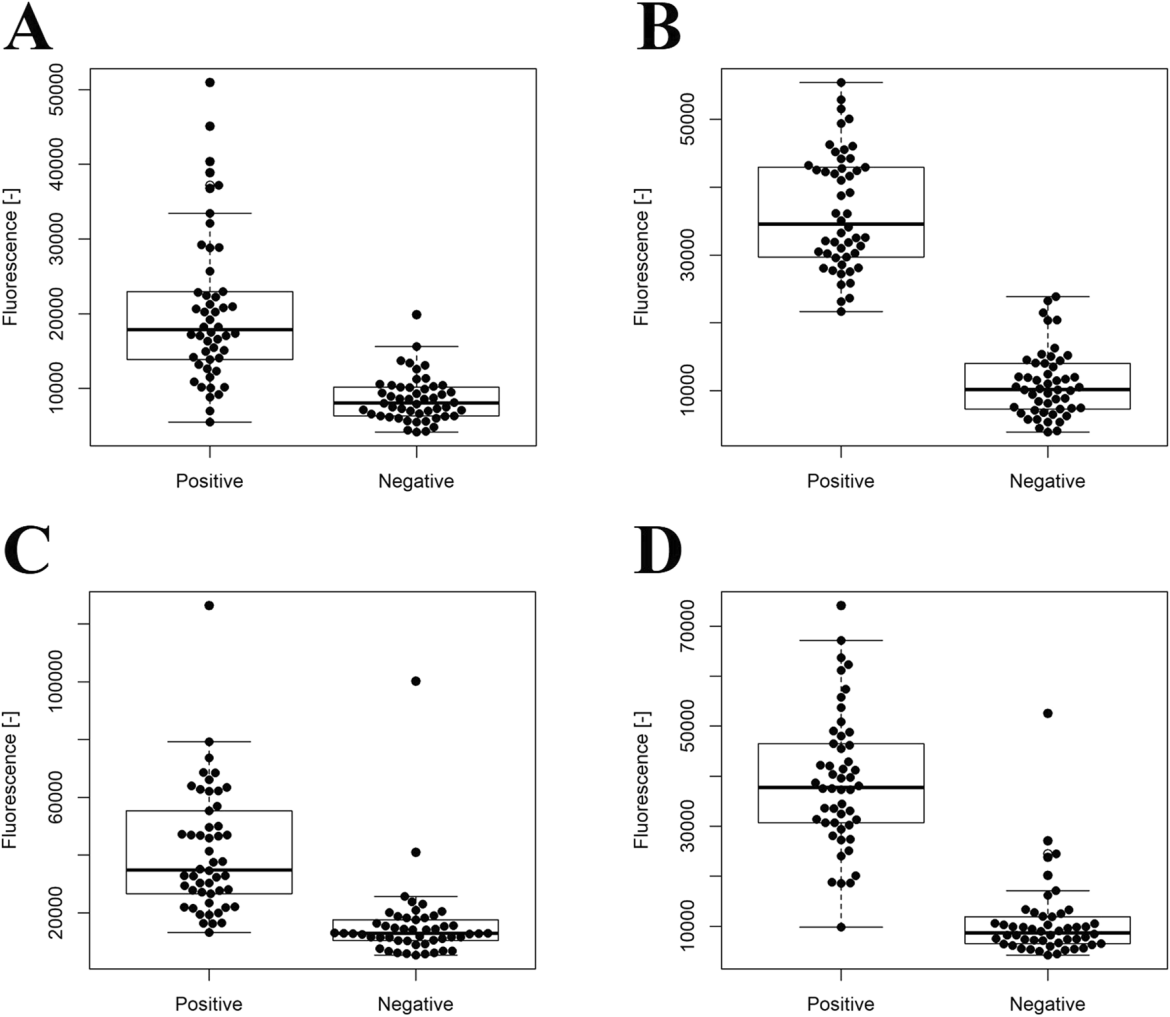
Bile acid binding activity of the top and bottom 50 peptides. In order to evaluate the 4-mer (**A**), 5-mer (**B**), 6-mer (**C**), and 7-mer (**D**) models, the 50 peptides predicted to have the most and least bile acid binding activity were synthesized, and their bile acid-binding activity was evaluated using a peptide array. In each group, 50 peptides were selected. The 50 peptides with the most predicted activity designated as ‘positive’, and the 50 peptides with the least predicted activity designated as ‘negative’.

All peptide sequences generated from edible proteins were applied to the acquired RF model. All applied peptides were labeled by output “probability”, since the RF model is a discrimination model. The results are shown in Supplementary Table S4. Applied peptides were ranked in order of probability, and the top 50 positive and bottom 50 negative predicted peptides were extracted. Those peptides were synthesized and their bile acid binding activities were determined using a peptide array. The synthesized sequences are listed in Supplementary Table S5, and their fluorescence intensities are shown in Fig. 4. The average fluorescence intensity of positive peptides was higher than that of negative peptides (P < 0.001), indicating that the RF model could successfully predict bile acid binding activity. Those probability values were also listed in Supplementary Table S5. Since those values were nearly 1.0, the correlation between theoretical and experimental parts could not be discussed precisely. The details of the peptides are shown in Supplementary Table S6.

**Figure 5:**
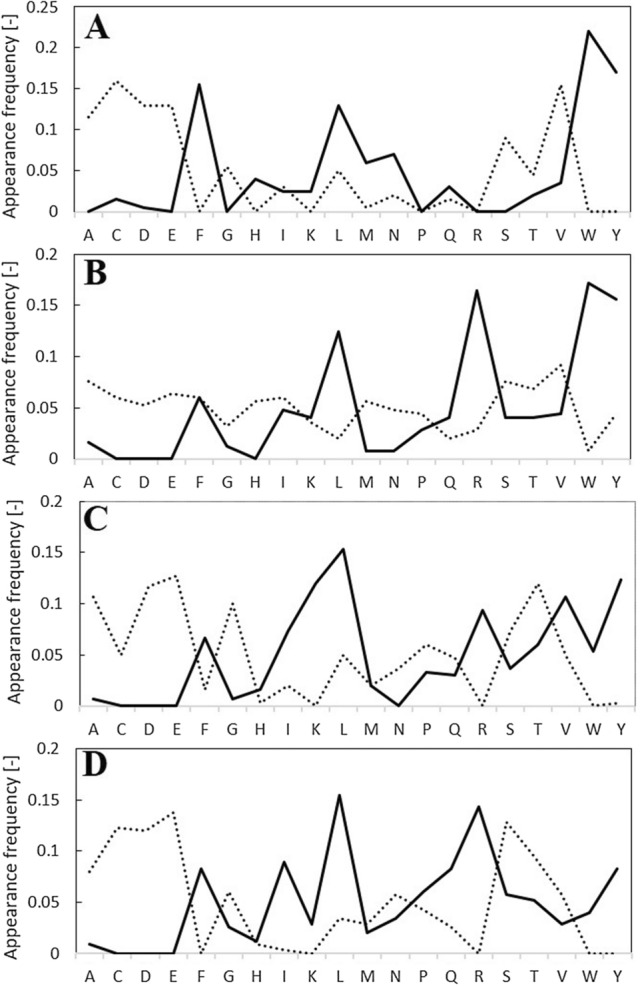
Appearance frequency of amino acid residues for top 50 (solid lines) and bottom 50 (dotted lines) peptides from predictive model [(**A**) 4-mer, (**B**) 5-mer, (**C**) 6-mer, (**D**) 7-mer].

We analyzed the appearance frequency of amino acid residues from Supplementary Table S5 and prepared Fig. 5 to verify the accuracy of the results of the predictive model. Since only 50 top or bottom peptides were used for appearance frequency, an explicit discussion was not clarified. However, 3 amino acids, F, L, and Y, showed a high frequency in positively predicted peptides, while W was high in 4-mer predicted peptides and R was high in 5-, 6-, and 7-mer predicted peptides. The different frequencies of positively predicted peptides may be due to the relatively low discrimination between positive and negative peptides (Fig. 4).

### Novel bile acid binding peptides from edible proteins

The top five peptides, ranked by fluorescence intensity in a peptide array for bile acid binding, are shown in Table 4. Seven of the peptides with the highest scores for bile acid-binding activity mapped to storage proteins in the database: VFWM from legumin A (*Pisum sativum*)^32^, QRIFW from high-molecular-weight glutenin (*Triticum aestivum*)^33^, RVWVQ from profilin-1 (*Hordeum vulgare*)^34^, LIRYTK from serum albumin (*Gallus gallus*)^34^, NGDEPL from legumin chain B fragment (*Vicia faba*)^35^, PTFTRKL from chicken connectin (titin) fragment (*Gallus gallus*)^34^, and KISQRYQ from alpha-S2-casein (*Bos taurus*)^34^. NGDEPL was predicted to have a low affinity for bile acid; however, it had a high bile acid-binding activity according to the peptide array. The mechanisms underlying this apparent contradiction are unclear, but this peptide might bind stereospecifically to bile acids. Since storage proteins are favorable for the manufacture of health foods and cosmetics, these protein sources are expected to contain novel bioactive components.

**Table 4:** The details of the top 5 peptides with the highest probability of having bile acid binding activity.

Sequence	Class	Protein	Position	Fluorescence intensity
**4-mer**				
MKWW	Positive	Beta-2 adrenergic receptor, rainbow trout (*Oncorhynchus mykiss*)	174_177	50,964
WWKW	Positive	Avenoindoline-a, precursor, oat (*Avena sativa*)	68_71	45,085
HWMW	Positive	Braching enzyme [Precursor], rice (*Oryza sativa*)	435_438	40,387
		Starch braching enzyme rbe4, rice (*Oryza sativa*)	450_453	
VFWM	Positive	Legumin A , precursor, garden pea (*Pisum sativum*)^a^	148_151	38,886
YMFK	Positive	Glyceraldehyde 3-phosphate dehydrogenase of rainbow trout (*Oncorhynchus mykiss*)	44_47	37,177
**5-mer**				
LWYRP	Positive	Secretory carrier membran protein, rice (*Oryza sativa*)	172_176	55,448
QRIFW	Positive	Glutenin, high molecular weight (HMW), precursor, wheat (*Triticum aestivum*), subunit DX5^a^	99_103	52,884
		Glutenin, high molecular weight (HMW), wheat (*Triticum aestivum*) subunit 1Dx2.1^a^	99_103	
		Glutenin, high molecular weight (HMW), wheat (*Triticum aestivum*) subunit (fragment)^a^	99_103	
		Glutenin, high molecular weight (HMW), wheat (*Triticum aestivum*)^a^	79_83	
AVRWP	Positive	Alpha-gliadin storage protein, wheat (*Triticum aestivum*)	20_24	51,521
RVWVQ	Positive	Profilin-1, barley (*Hordeum vulgare*)^a^	31_35	50,055
GWRSY	Positive	Glucocorticoid receptor, rainbow trout (*Oncorhynchus mykiss*)	584_588	49,414
**6-mer**				
LIRYTK	Positive	Serum albumin, precursor, chicken (*Gallus gallus*)^a^	436_441	126,399
NGDEPL	Negative	Legumin chain B fragment, broad bean (*Vicia faba*)^a^	9_14	100,178
VIYRLK	Positive	Probable cell division protein ftsW	63_68	79,195
KLFTKT	Positive	Germin-like protein 4, rice (*Oryza sativa*)	135_140	73,601
IYRLKL	Positive	Probable cell division protein ftsW	64_69	68,634
**7-mer**				
KFMYRSG	Positive	Paramyosin (*Sarcoptes scabiei*) (Q9BMM8)	7_13	74,124
LKIRYSS	Positive	Starch debranching enzyme,rice (*Oryza sativa*)	865_871	67,116
		Starch debranching enzyme [Precursor],rice (*Oryza sativa*)	863_869	
PTFTRKL	Positive	Chicken connectin (titin), fragment (*Gallus gallus*)^a^	469_475	63,712
KISQRYQ	Positive	Alpha-S2-casein gen. var. A, precursor, bovine (*Bos taurus*)^a^	181_187	62,301
		Alpha S2-casein gen. var. C, fragment (16-222), bovine (*Bos taurus*)^a^	166_172	
		Alpha S2-casein gen. var. D, fragment (16-213), bovine (*Bos taurus*)^a^	157_163	
RQFMKSL	Positive	Lactoferrin binding protein A precursor (*Neisseria meningitidis*)	203_209	61,136
		Lactoferrin receptor (*Neisseria gonorrhoeae*)	203_209	
		Lactoferrin binding protein (*Neisseria meningitidis*)	199_205	

^a^Storage proteins. Protein refers to the parent proteins and position refers to the site of the peptides from the N-terminus in the BIOPEP-UWM database.

Most of the predicted BPs in the present dataset were obtained by proteolysis by enzymes from plants or microorganisms and proteolysis by gastrointestinal enzymes^36^. Therefore, to evaluate the utility of these peptides at the industrial scale, we examined whether the seven peptides derived from storage proteins could be generated using peptidases or proteases. As a result, KISQRYQ was predicted to be generated from alpha-S2-casein (*Bos taurus*) with peptidyl-Lys metalloendopeptidase (*Armillaria mellea* neutral proteinase). Gutiez et al. previously investigated the relationship between autolysis caused by lactic acid bacteria and the production of angiotensin-converting enzyme (ACE)-inhibitory peptides, and reported that KISQRYQ was generated from skimmed milk (alpha-S2-casein) by *Lactococcus lactis subsp. lactis IL1403*^37^. Taken together, these results suggest that KISQRYQ could be a candidate BP for health food.

In the present study, a new BP screening method was developed based on a synthetic peptide library for bile acid binding and machine learning. A database containing peptide sequences derived from edible proteins was developed to identify peptides with features associated with bile acid binding. Novel bile acid-binding candidate peptides were discovered by combining these two tools. Among the peptides with the highest predicted scores for bile acid binding activity, seven (VFWM, QRIFW, RVWVQ, LIRYTK, NGDEPL, PTFTRKL, and KISQRYQ) were derived from storage proteins. Among them, KISQRYQ was predicted to be generated from alpha-S2-casein (*Bos taurus*) with peptidyl-Lys metalloendopeptidase (*Armillaria mellea* neutral proteinase) or from skim milk with *Lactococcus lactis subsp. lactis IL1403*. Our novel method could successfully screen BPs and can be easily applied to industrial applications based on whole edible proteins. The proposed approach would be useful for bile acid-binding peptides, as well as for other BPs, provided that a large amount of training data can be obtained.

## Materials and methods

### Materials

The Fmoc amino acid OH was purchased from Watanabe Chemical Industries, Ltd. (Japan). BSA was purchased from Fujifilm Wako Pure Chemical Corporation (Japan). Taurocholic acid (T-4009) was purchased from Sigma-Aldrich (USA). The anti-cholic acid antibody (FKA502) was purchased from Cosmo Bio (Japan). Anti-rabbit IgG-conjugated Alexa 488 (ab150077) antibody was purchased from Abcam (Cambridge, UK).

### Synthetic peptide array generation

To generate positive and negative peptide training datasets for our machine learning algorithm, we synthesized 460 4-, 5-, 6-, and 7-mer peptides that were randomly generated using R software (version 3.5.3) (R development Core Team, https://www.r-project.org/). All peptides were synthesized on a cellulose membrane with a spot synthesizer (Intervis, ASP222, Cologne, Germany), as previously reported^38^. Fmoc-aund-OH was introduced at the C-terminal end of the peptide as a spacer. After synthesis, the side-chain-protecting groups of the Fmoc amino acids were removed using trifluoroacetic acid. The membrane was washed thoroughly with diethyl ether and methanol and dried. The membrane was soaked in PBS for 24 h and then transferred into 1% BSA in PBS solution at 37 °C for 12 h before the assay commenced.

### Bile acid binding assay

A bile acid-binding assay was conducted according to a previous study^23^. After washing the peptide array with PBS, 10 μg/mL taurocholic acid dissolved in PBS was added to the arrays and incubated for 1 h. After washing with PBS, anti-cholic acid antibody dissolved in 0.25% BSA was added to the array and incubated for 1 h at 37 °C. After washing with TBS containing 0.05% Tween 20, 2 μg/mL of anti-rabbit IgG conjugated to Alexa 488 dissolved in PBS was added and incubated for 1 h at 37 °C. After washing with TBS, peptide spots were fluorescently detected using a fluorescent imager (Typhoon FLA-7000, GE Healthcare Japan Life Sciences, Tokyo, Japan). The scanned images were quantified using Image Quant TL (GE Healthcare Japan Life Sciences, Tokyo, Japan). Average fluorescence intensities were calculated by subtracting the peptide array treated only with the secondary antibody from the triplicate fluorescence intensities of the same peptide sequence.

### Feature generation

Seven features were considered for the prediction of bile acid binding activity (Supplementary Table S1). General physicochemical features of peptides were described by pI^26^, polarity^26^, hydrophobicity^39^, and molecular weight^27^, while structural features were described by Ph (the index about helix) and Pt (the index about turn). Xia et al. investigated the existence of amino acids in secondary structures and defined new indices, Ph, Ps (the index of the sheet), and Pt^40^. The correlation coefficient between Ph and Ps was > |0.98|; therefore, Ps was excluded from the feature index in this study. In addition, previous research has revealed that hydrophobic amino acids, especially aromatic ones, interact with bile acid micelles^19,30,31,41^. Therefore, the number of aromatic amino acids was included as a peptide feature. Based on these features, the GFs of the library peptides were generated. For example, in the case of 4-mer peptides, each amino acid has seven features (Supplementary Table S1), and 28 AAFs were also generated for each 4-mer peptide. In addition, four global values, the maximum, minimum, average, and standard deviation (sd) were generated for each peptide. This means that a total of 56 features (28 AAFs and 28 GFs) were generated and used as explanatory variables for each 4-mer peptide. All features were calculated in R.

### Construction of prediction models

To construct the prediction model, three algorithms were used: support vector machine (SVM), random forest (RF)^42^, and logistic regression (LR). Scikit-learn libraries^43^ were adopted, and leave-one-out cross-validation (LOOCV) was imported into Python. The parameters for the algorithms were set as follows: In the SVM (linear) model, the default value of the parameter cost (C = 1) was used. In the RF model, the number of trees to grow (n_tree_) were set at 100 or 500, and the number of variables randomly sampled as candidates at each split (m_try_) was set to “auto.” In the LR model, the penalty was set to “lasso,” C was set to 10 or 50, and the maximum number of iterations required for the solvers to converge (max_iter) was set to 100. The probability of binding to bile acid was calculated for all peptides and classified based on a score of 0.5.

The performance of all three machine learning models was evaluated using 3 metrics:

$${\text{Accuracy }} = \, \left( {{\text{TP }} + {\text{ TN}}} \right)/\left( {{\text{TP}} + {\text{TN}} + {\text{FP}} + {\text{FN}}} \right),$$

$${\text{Precision }} = \, \left( {{\text{TP}}} \right)/\left( {{\text{TP}} + {\text{FP}}} \right),$$

$${\text{Recall }} = \, \left( {{\text{TP}}} \right)/\left( {{\text{TP}} + {\text{FN}}} \right).$$

TP is the true positive; TN is the true negative; FP is the false positive; FN is the false negative.

### Generation of peptide database for edible proteins

A total of 710 protein sequences were obtained from BIOPEP-UWM, available at http://www.uwm.edu.pl/biochemia/index.php/pL/biopep (accessed in October 2018)^11^. Peptides were generated based on the entire sequence of proteins. All sequences were sectioned into 4-, 5-, 6-, and 7-mer peptide fragments with one residue shift from the N-terminal amino acid. The peptide database was generated in csv format. For cleavage site prediction, PeptideCutter available at https://web.expasy.org/peptide_cutter/ was used^44^ and all enzymes stored in the software were used as simulation enzymes.

### Statistical analysis

Data are presented as means ± standard deviation (SD). Student’s t-test was used for between-group comparisons. Statistical significance was set at p < 0.05.

## Supplementary Information

Supplementary Figures.

Supplementary Tables.
