# Supplementary material for: Machine learning screening of bile acid-binding peptides in a peptide database derived from food proteins
Source: Sci Rep. 2021 Aug 9;11:16123. doi: 10.1038/s41598-021-95461-1 (PMC8352859; doi:10.1038/s41598-021-95461-1)
Supplement: Supplementary file 1 — Supplementary Figures. [file 41598_2021_95461_MOESM1_ESM.docx]

**Machine learning screening of bile acid-binding peptides in a peptide database derived from food proteins**

*Kento Imai^1,2^, Kazunori Shimizu^1^, Hiroyuki Honda^1^**

^1^Department of Biomolecular Engineering, Graduate School of Engineering, Nagoya University, Nagoya 464-8603, Japan

^2^Japan Society for the Promotion of Science, Research Fellowship for Young Scientists, Chiyoda-ku, Tokyo, Japan


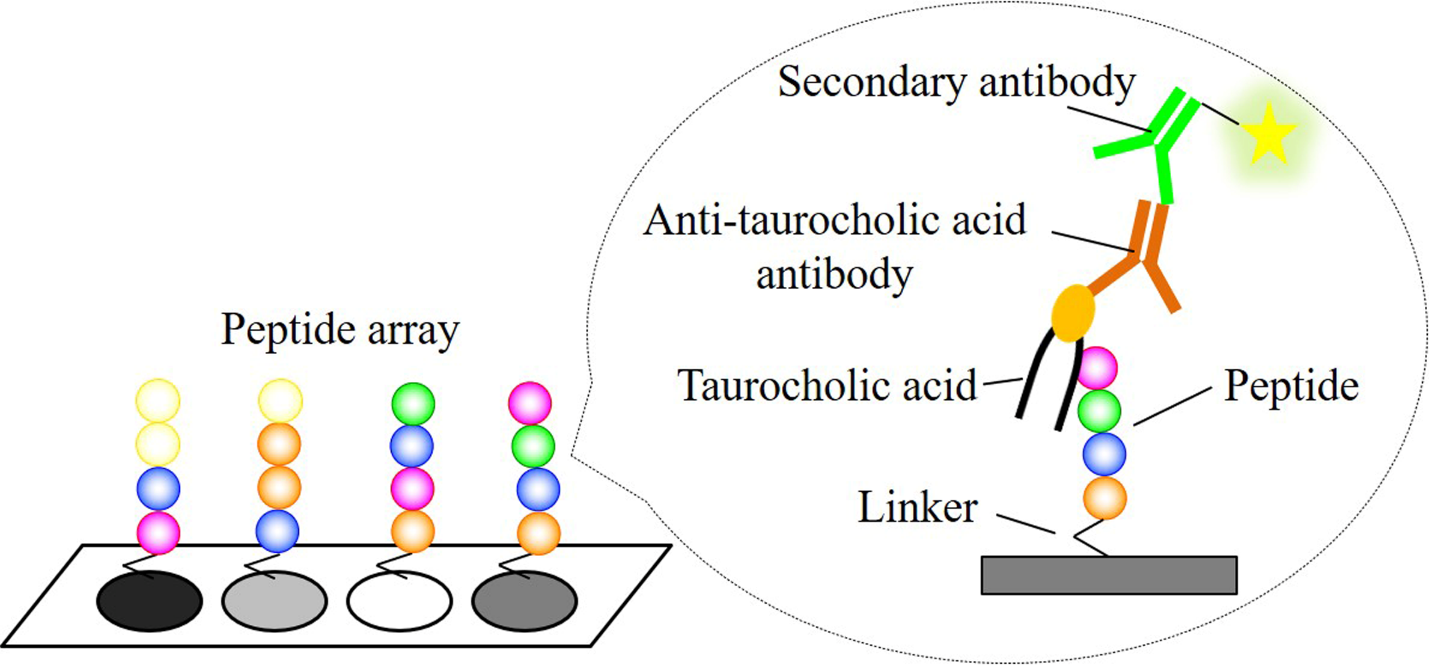


**Figure S1: Assessment of bile acid binding activities using peptide array**


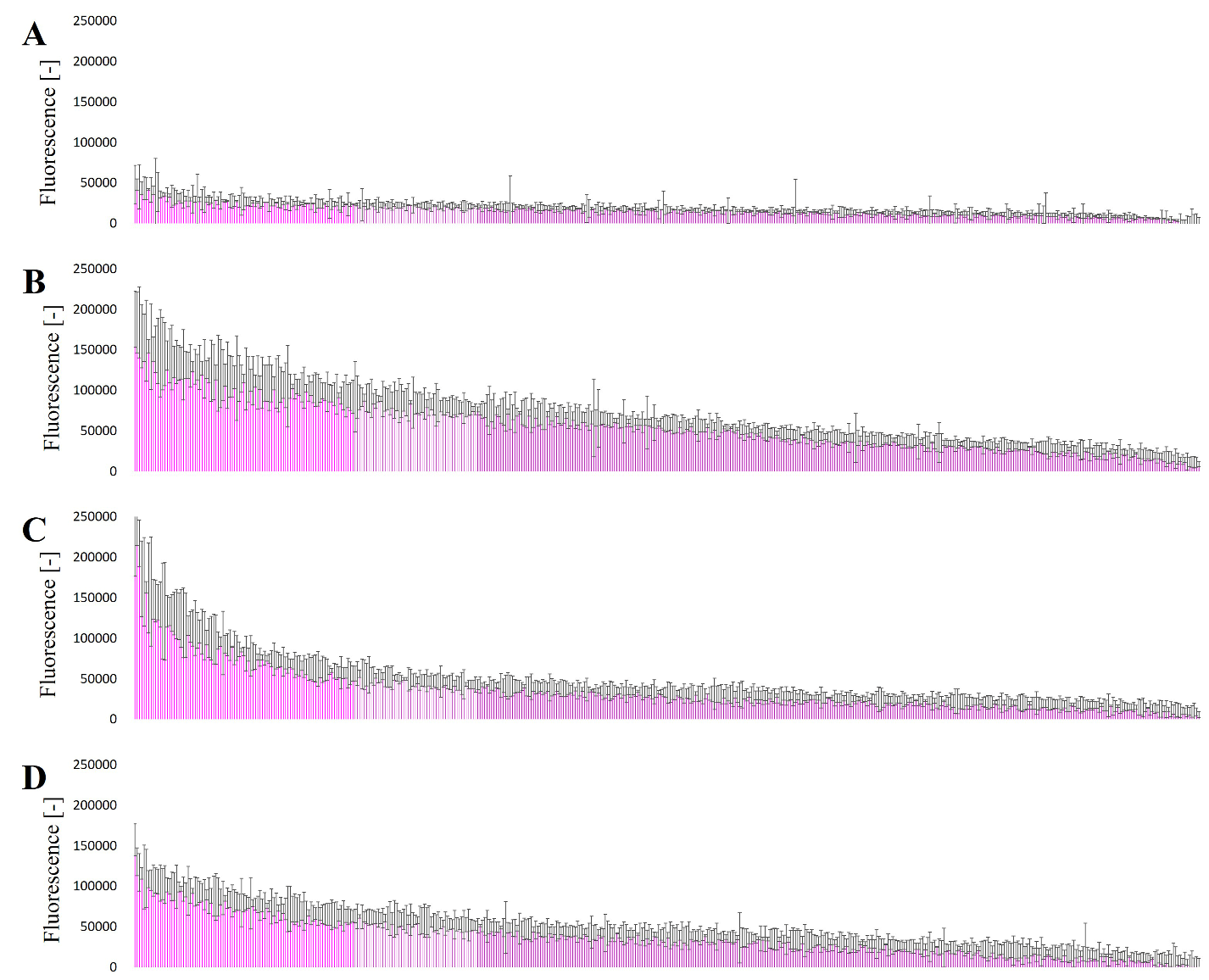


**Figure S2: The fluorescent intensities for the training datasets according to a peptide array to detect acid bile binding activity.**

The intensities are shown for 4-mer (A), 5-mer (B), 6-mer (C), and 7-mer (D) synthetic peptides.

**Table S1: Features used to construct the model.**

**Table S2: All florescence intensities used to generate training datasets.**

**Table S3: Selected features of the RF prediction model for bile acid binding activity.**

**Bold type**: features related to molecular weight, underlined: features related to isoelectric point, **both bold type and underlined**: features related to ‘aromatic amino acids’.

**Table S4: The number of peptides that were predicted as positive or negative for bile acid binding activity in the database.**

**Table S5: Sequences of peptides that were synthesized for the evaluation of the model.**

P-positive and N-negative

**Table S6: The details of the peptides synthesized in Table S5**
